# ASO Author Reflections: Are Tumor-Infiltrating Neutrophils Drivers of Metastatic Disease in Esophageal Cancer Patients Treated with Neoadjuvant Therapy?

**DOI:** 10.1245/s10434-022-12578-x

**Published:** 2022-09-29

**Authors:** Carlos S. Cabalag, Wayne A. Phillips, Cuong P. Duong, Nicholas J. Clemons

**Affiliations:** 1 Cancer Research, Peter MacCallum Cancer Centre, Melbourne, VIC Australia; 2 Cancer Surgery, Peter MacCallum Cancer Centre, Melbourne, VIC Australia; 3 Sir Peter MacCallum Department of Oncology, University of Melbourne, Parkville, VIC Australia; 4 Department of Surgery (St. Vincent’s Hospital), University of Melbourne, Fitzroy, VIC Australia

## Past

The mainstay of treatment for patients with locally advanced esophageal cancer is neoadjuvant chemotherapy, with or without radiotherapy, followed by surgical resection. Neoadjuvant treatment affects the tumor immune microenvironment (TME), which is known to play a significant role in tumor regression or proliferation.^1^ Earlier studies demonstrated that chemotherapy and radiotherapy exerts a summative immuno-stimulatory effect mediated by the DNA damage response, tumor cell death, presentation of neoantigens, and subsequent activation of the adaptive immune response.^2,3^ Nevertheless, emerging evidence suggests that neoadjuvant treatment of tumors with a chronically inflamed TME may actually result in an immune-suppressive microenvironment mediated by neutrophils.^4^

## Present

Esophageal cancers arise from chronically inflamed microenvironments, and little is known about the effect of neoadjuvant treatment on the esophageal TME. This study^5^ is the first to demonstrate in esophageal cancer that neoadjuvant treatment upregulates genes related to myeloid cell differentiation and that high numbers of tumor-infiltrating CD15^+^ neutrophils are associated with early recurrence of disease. These findings identify tumor-infiltrating neutrophils as a possible mechanism of “immune escape” responsible for early recurrence of esophageal cancer. Furthermore, this study verified, using an independent cohort, that the magnitude of neutrophil infiltration is a biomarker for early disease recurrence that is more prognostic than pathologic lymph node status or tumor regression grade.

## Future

The aforementioned critical finding opens up a new therapeutic window to target neutrophils and their interaction with the TME to mitigate the risk of tumor cell dissemination in esophageal cancer. However, further work is required to delve deeper into the mechanistic aspects of how neutrophils mediate their pro-tumorigenic effects.
